# A FRET-based cGMP biosensor in *Drosophila*

**DOI:** 10.17912/micropub.biology.000887

**Published:** 2023-11-28

**Authors:** Ana Clara Gonzalez, Cecilia Abreu, Sergio Pantano, Marcelo Comini, Leonel Malacrida, Boris Egger, Rafael Cantera, Daniel Prieto

**Affiliations:** 1 Departamento de Biología del Neurodesarrollo, Instituto de Investigaciones Biológicas Clemente Estable, Montevideo, Montevideo, Uruguay; 2 Molecular, Cellular and Animal Technology Program, Institut Pasteur de Montevideo, Montevideo, Montevideo, Uruguay; 3 BioMolecular Simulation Group, Institut Pasteur de Montevideo, Montevideo, Montevideo, Uruguay; 4 Redox Biology of Trypanosomes Lab, Institut Pasteur de Montevideo, Montevideo, Montevideo, Uruguay; 5 Advanced Bioimaging Unit, Institut Pasteur de Montevideo, Montevideo, Montevideo, Uruguay; 6 Universidad de la República, Montevideo, Montevideo, Uruguay; 7 Department of Biology, University of Fribourg, Fribourg, Fribourg, Switzerland; 8 Departamento de Neurofisiología Celular y Molecular, Instituto de Investigaciones Biológicas Clemente Estable, Montevideo, Montevideo, Uruguay

## Abstract

CUTie2 is a FRET-based cGMP biosensor tested so far only in cells. To expand its use to multicellular organisms we generated two transgenic *Drosophila melanogaster* strains that express the biosensor in a tissue-dependent manner. CUTie2 expression and subcellular localization was verified by confocal microscopy. The performance of CUTie2 was analyzed on dissected larval brains by hyperspectral microscopy and flow cytometry. Both approaches confirmed its responsivity, and the latter showed a rapid and reversible change in the fluorescence of the FRET acceptor upon cGMP treatment. This validated reporter system may prove valuable for studying cGMP signaling at organismal level.

**Figure 1.**
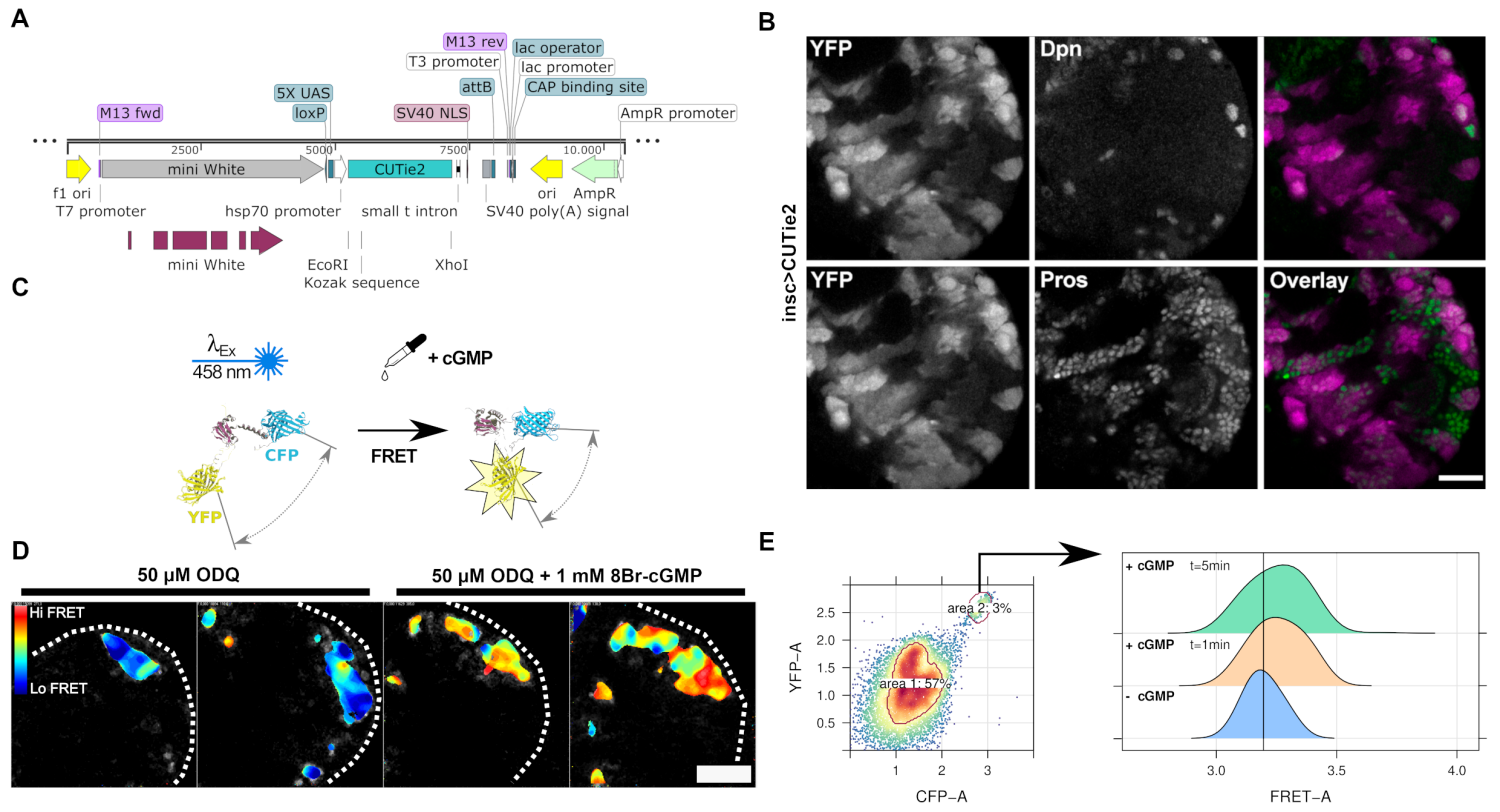
A. Schematic representation of the construct used to introduce the CUTie2 biosensor under UAS control in flies; B. Single confocal plane of the left hemisphere of a larval brain of the *insc-GAL4 x UAS-CUTie2* F1 marked with anti-Dpn (top) with an AlexaFluor™568-conjugated anti-guinea pig secondary antibody or anti-Pros (bottom) with Cy5-conjugated anti-mouse secondary antibody to stain neuroblasts and ganglion mother cells (green), respectively and the YFP module of CUTie2; C. Schematic depicting the allosteric change of CUTie2 upon exposure to cGMP. Fluorescent modules get close enough for FRET to occur under CFP excitation with 458nm light. D. Hyperspectral FRET images of CUTie2 in the larval brain neuroepithelium. Dissected brains from GAL4 ^c855a^ x UAS-CUTie2 F1 were incubated for 10 min with 5 µM ODQ to block endogenous soluble guanylyl cyclases and imaged. Then, 1 mM 8Br-cGMP was added and imaged after 1 min. The color-coded images were generated by spectral phasor analysis. E. Response of CUTie2 to cGMP. Flow cytometry analysis of disaggregated brain cells from *insc-GAL4 x UAS-CUTie2* F1 brains upon addition of 100 μM 8Br-cGMP. The scatter plot shows the data-driven gate (area 2) used for selection of CFP+/YFP+ cells. The monoparametric density plots depict their fluorescence intensity on the FRET channel before and after 1 min and 5 min from the addition of 8Br-cGMP. The black vertical line indicates the median fluorescence intensity of these cells without addition of 8Br-cGMP. Scale bar: 20µm.

## Description

Neural stem cells (NSCs) are necessary for both embryonic and adult neurogenesis. Their activity and fate are regulated in part by the environment of the local niche where they reside (Engler et al., 2018) . Several studies have shown that oxygen levels can influence this environment, promoting the development and differentiation of certain cell types or inhibiting others (Simon & Keith, 2008) . It has been shown that in the larval brain of *D. melanogaster* , a major region rich in stem cells (the optic lobe), receives very few tracheoles (respiratory tubes) and is hypoxic relative to the central brain, which contains mainly active neurons and is penetrated by a large number of tracheoles (Baccino-Calace et al., 2020; Hofbauer & Campos-Ortega, 1990) . Several mechanisms regulate oxygen levels in the body and the most studied is the one based on the hypoxia-inducible factor (HIF) (Semenza, 2012) . Activation of this pathway is accompanied by nuclear accumulation of HIF/Sima (Romero et al., 2007) , lactate dehydrogenase expression (Lavista-llanos et al., 2002), and tracheolar growth (Centanin et al., 2008; Jarecki et al., 1999) . However, none of these three key features was observed in the optic lobe of *D. melanogaster* larval brains (Baccino-Calace et al., 2020; Misra et al., 2017) . It has also been shown both in *D. melanogaster* and other invertebrates that the subunits of atypical soluble guanylate cyclase (asGC) function as a molecular oxygen sensor by binding to a heme group (Morton, 2004) . A first study of the spatial expression pattern of asGCs based on *in situ* hybridization, showed they are expressed within the larval *Drosophila* brain (Langlais et al., 2004) .

Here we report the building of fly stocks that express the cGMP biosensor called CUTie2 (Klein et al., 2021) . CUTie2 is an allosteric biosensor that consist of a protein kinase GI (PKGI) binding site with high affinity for cGMP and a pair of fluorophores (CFP and YFP) whose excitation/emission spectra are compatible with Föster resonance energy transfer (FRET; Klein et al., 2021). cGMP binding by the unfolded region of PKGI triggers a conformational change that approaches both fluorescent modules allowing FRET to occur ( Figure 1C ). The recombinant form of the biosensor was characterized *in vitro* and proved highly sensitive and selective for detecting sub-μM concentrations of cGMP (Klein et al., 2021) .

We cloned the DNA encoding CUTie2 into a pUAST vector to generate transgenic strains of *D. melanogaster* that express the biosensor under the control of a UAS element ( Figure 1A ). This will make possible to obtain targeted expression of the sensor using the UAS-GAL4 system (Brand & Perrimon, 1993) . We generated a strain with the CUTie2 biosensor on chromosome II marked with the CyO balancer (;UAS-CUTie2/CyO) and a strain with the biosensor on chromosome III marked with the TM3 balancer (;;UAS-CUTie2/TM3). By crossing both strains, we obtained the homozygous larvae used for the experiments.

To confirm that using the UAS-GAL4 system can be expressed in different cell types we examined the fluorescence emitted by the biosensor in brains derived from crosses between flies carrying the biosensor and flies with drivers specific for different cell types or tissues. As fixation of the samples greatly reduced the CFP fluorescence, we examined ex vivo non-fixed brains. Confocal laser microscopy confirmed that it is possible to direct the expression of the biosensor to specific cell types. Double staining with antibodies specific for either neuroblast (anti-Deadpan) or ganglion mother cells (anti-Prospero) confirmed that the sensor was expressed in both cell types in the brain of larvae from the cross *insc-GAL4 x UAS-CUTie2* ( Figure 1B ).

Using hyperspectral microscopy and spectral phasor analysis (Torrado et al., 2022) we validated the sensor’s responsivity to cGMP *ex vivo* . Brains of c855a-GAL4 x UAS-CUTie2 larvae were incubated with the soluble guanylyl cyclase inhibitor ODQ (50 µM) for 5 minutes to reduce spurious cGMP signal. Then, as a proof-of-principle a high concentration of the cell-permeant cGMP-analog 8-Br-cGMP was added and the brains were imaged after 1 minute. With this approach we were able to identify an increase in the FRET signal ( Figure 1D ).

The changes caused by the addition of the cGMP-derivative in the FRET-channel intensity within CFP-, YFP-positive cell populations were also investigated by flow cytometry in samples from disaggregated brains from the cross *insc-GAL4 x UAS-CUTie2* . Addition of the cGMP derivative caused increase in the intensity of the FRET emission one minute after adding the ligand compared with the untreated control. This increase was maintained for at least 5 minutes ( Figure 1E ).

The strains generated in this project are available to the scientific community. They are kept at the IIBCE Uruguay, and in the Department of Biology of the University of Fribourg, Switzerland.

## Methods

*Generation of genetic constructs:* We used restriction-free cloning (van den Ent & Löwe, 2006) with primers designed to amplify the biosensor sequence in a first reaction, and then amplified the plasmid with the said sequence in a subsequent reaction. Primers for the insertion of attB sites into the pUAST vector and a plasmid containing the sequence of the CUTie2 biosensor (Klein et al., 2021) were used. The PCR product was incubated for 90 minutes at 37°C with *Dpn* I to eliminate the methylated parental plasmid, and then for 20 minutes at 80°C to inactivate the enzyme. The product was used to transform chemically competent *Escherichia coli* and the plasmid was purified by midiprep columns (Qiagen).

*Generation of D. melanogaster transgenic strains:* The plasmid containing the biosensor was sent to BestGene Inc, where it was injected into *D. melanogaster* zygotes. Using PhiC31 integration, we inserted the transgene at a second chromosome landing site located at approximate cytolocation 25C6 onto an attP40 stock. For the chromosome III, UAS-CUTie2 stock the approximate cytolocation was 68A4 onto a 8622 BDSC stock. We generated a homozygous UAS-CUTie2 (II) stock through standard mating, which was used for all the experiments.

*Fly maintenance:* Flies were raised on standard cornmeal medium at 25°C 12:12 h light:darkness cycles as previously described (Baccino-Calace et al., 2020) .

*Targeting of the biosensor using the GAL4-UAS system:* Using expression targeting with the UAS-GAL4 system (Brand & Perrimon, 1993) we generated larvae that expressed the CUTie2 biosensor in the optic lobe or neural stem cells using the driver *insc-GAL4 and GAL4 ^c855a^* .

*Fluorescence detection by flow cytometry:* Twenty-five brains of wandering third-instar larvae from *insc-GAL4 x UAS-CUTie2* F1 and 25 of the UAS-CUTie2 parental line were dissected. They were washed three times with PBS and incubated for one hour at room temperature with collagenase I in Rinaldini buffer at a final concentration of 0.5 mg/ml as previously described (Egger et al., 2013) and centrifuged at 300 *g* . The supernatant was removed, and the pellet was washed and resuspended in PBS. Samples were filtered (40 µm) to remove cell clumps before acquisition in an Attunne Nxt cytometer (Thermo Fisher Scientific). A solution of 8Br-cGMP (Tocris) was added to a final concentration of 100 μM. Briefly, anomalies were identified and subtracted using flowAI (Monaco et al, 2016) and data-driven gates were used to select the cell population and debris excluded using FSC vs. SSC, select singlets and CFP/YFP double-positive cells. Monoparametric analysis of the FRET channel performed on double-positive cells. The physical filters used were BL1 for YFP (excitation: 488nm; emission: 530/30nm), VL1 for CFP (ex: 405nm; em: 440/50nm) and VL2 for FRET (ex: 405nm; em: 512/25nm). The FRET channel shows YFP emission upon excitation of CFP. Direct excitation of the acceptor was not subtracted, nor any ratio calculation performed. Gating strategy and data analyses were performed in R-Bioconductor, using an R script developed by DP ( https://github.com/danielprieto/Gonzalez_etal_2023 ).

*Detection and analysis using confocal microscopy:* Brains of wandering third-instar larvae from each cross ( *UAS-CUTie2* crossed to *insc-GAL4* ) were dissected and fixed in 4% paraformaldehyde in PBS for 10 minutes. The samples were washed three times with PBS containing 0.5% v/v Triton X-100 (PBS-T) and incubated overnight at 4°C with primary antibodies. The following day, the samples were washed three times with PBS-T and incubated with the corresponding secondary antibodies overnight at 4ºC, washed twice with PBS-T and one last time with PBS before mounting in 80% v/v glycerol in 0.1 M Tris pH=8.8.

Imaging was performed with a ZEISS LSM 800 microscope using 20X (NA=0.8), and 63X (AN=1.4) objectives. YFP was excitated with a 488nm laser line and CFP with 405nm. Images were processed for display with ImageJ used the median filter with a radius of 1.2 pixels. Hyperspectral imaging was performed using a ZEISS LSM 880 microscope under a 63X (NA=1.4) water immersion objective using a 458nm laser line for CFP excitation. Spectral data was acquired in 31 10nm windows ranging from 413 to 713 nm at 256x256 pixels. FRET states were identified as the linear combination of the CFP and YFP modules on the spectral phasor plot and a heat map LUT applied onto the reciprocal mean intensity display images. HSI data was analyzed using spectral phasor approach using SimFCS software (Globals for Images, G-Soft Inc.).

## Reagents

*Primers*

**Table:** 

*Name*	*Description (5’→3’)*
pUASTattB_GMPc_Fw	TCTGAATAGGGAATTGGGAATTCGTGAATTCATGCGGACCGGACTGATCAAACATA
pUASTattB_GMPc_Rv	ATACAGTTCATCCATCCCCAGGTAACTCGAGAAAATACAGTTCATCCATCCCCAGG

*Fly stocks*

**Table:** 

*Name*	*Description*
UAS-CUTie2 chr. II	y1w67c23; P{CaryP}attP40[W.UAS-CUTie2]/ P{CaryP}attP40[W.UAS-CUTie2]
UAS-CUTie2 chr. III	y1w67c23; ;P{CaryP}attP2[W.UAS-CUTie2]/ P{CaryP}attP2[W.UAS-CUTie2]
inscuteable-GAL4	BDSC #8751
GAL4 ^c855a^	BDSC #6990

*Antibodies*

**Table:** 

*Description*	*Dilution*	*Source*
Guinea pig anti-Deadpan (Dpn)	1:2500	Kind gift from Prof. Jürgen Knoblich, Institute of Molecular Biotechnology, Vienna, Austria
Mouse anti-Prospero	1:10	MR1A, Developmental Studies Hybridoma Bank
AlexaFluor™568-conjugated goat anti-guinea pig	1:1000	Molecular Probes
Cy5-conjugated goat anti-mouse	1:1000	Molecular Probes
